# Fatal Infection of a Pet Monkey with *Human herpesvirus 1*

**DOI:** 10.3201/eid0806.010341

**Published:** 2002-06

**Authors:** Hartwig P. Huemer, Clara Larcher, Thomas Czedik-Eysenberg, Norbert Nowotny, Martin Reifinger

**Affiliations:** * University of Innsbruck, Innsbruck, Austria; † Tierklinik Rodaun, Vienna, Austria; ‡ University of Veterinary Medicine, Vienna, Austria; § United Arab Emirates University, Al Ain, United Arab Emirates

**Keywords:** primate, marmoset, Callithrix, Human herpesvirus, HHV-1, zoonosis

## Abstract

Concerns have been raised about pet monkeys as a potential threat to humans. We report the opposite situation, a danger to pets that arises from humans. Similar to herpesvirus B *(Cercopithecine herpesvirus 1),* which endangers humans but not its host species, *Human herpesvirus 1* can act as a “killer virus” when crossing the species barrier to New World monkeys.

A man was bitten by a marmoset (genus *Callithrix)* that had stomatitis. For exclusion of possible zoonotic pathogens, virus culture was performed on a specimen obtained from the marmoset’s oral mucosa. Virus isolation and typing with antibodies revealed *Human herpesvirus 1* (HHV-1) infection, confirmed by type-specific polymerase chain reaction (PCR). Despite treatment, the monkey died 2 days after the sample was drawn. Standard veterinary practice is to consider whether diseases of primates that have been in close contact with humans might have been caused by human viruses. Acute stomatitis in pet monkeys can suggest HHV-1 infection, among other diseases, and systemic treatment with acyclovir may be appropriate.

## Case Report

A 2-year-old male marmoset (*Callithrix jacchus)* was brought to a veterinary clinic with a 6-day history of severe necrotizing stomatitis, vomiting, and loss of appetite. The pet had been acquired by its owner 9 months earlier from an unknown source. Since then, it had usually been in close contact with its owner; she kept the pet on a leash and carried it directly on her body. A few days before being seen at the clinic, the animal had bitten a male visitor’s hand. Treatment of the marmoset included removal of the necrotic mucosal surface under anesthesia, local administration of acyclovir, and systemic application of antiemetic, antiphlogistic, and antibiotic agents. For diagnosis and exclusion of a possible zoonotic infection, a few samples of the altered oral mucosa were taken. Two days after veterinary intervention, the marmoset died. The owner refused a necropsy. Since communication with the owner ceased before diagnosis, questions about possible herpetic lesions on her or her guest who had been bitten by the monkey could not be answered.

One specimen of the mucosal membrane was fixed in 10% buffered formalin, dehydrated in ethanol, cut into 4-μm sections, and stained with hematoxylin and eosin. Histologic examination showed severe necrotizing stomatitis with purulent inflammation and bacterial colonization of the debris. No epithelium remained nor any morphologically visible indication of a specific infection.

Another specimen of the oral mucosa was homogenized in sterile phosphate-buffered saline, and the supernatant was used for cell culture and PCR analysis. Virus culture was performed on Vero cells originating from African green monkey kidney tissue (ATCC # CCL-81). One to two days later, a typical cytopathic effect was visible, consisting of plaques and cell rounding (Figure), which led to total detachment of the cells within 3 to 4 days.

**Figure:**
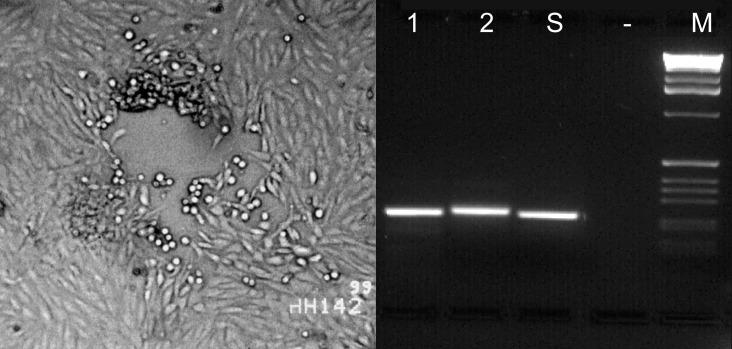
Left: cytopathic effect in Vero cells consisting of a plaque and rounding of the cells after homogenized altered mucosal membrane of the marmoset was added to the cell culture. Right: type-specific polymerase chain reaction (PCR). Lanes 1 and 2 show fragments of 229-bp DNA amplified from *Human herpesvirus 1* (HHV-1) and 241 bp from HHV-2 control strains, respectively. Lane S shows an HHV-1–specific PCR product amplified from an oral mucosa specimen of the marmoset; no product was obtained from supernatants of uninfected cell culture (lane -). Lane M, 1 kb DNA Ladder (GIBCO/BRL,Grand Island, NY).

Cells were fixed with acetone/methanol, and immunofluorescence staining was carried out by using monoclonal and polyclonal antibodies against different species of *Alphaherpesvirinae,* including HHV-1 and -2, suid (SuHV-1), equid (EHV-1 to -4), bovine (BoHV-1), and nonhuman primate (CeHV-1) viruses.

Positive staining was obtained with several monoclonal anti-HHV-1 antibodies directed against major glycoproteins (gC, gD, gE) as well as nonstructural proteins (infectious cell protein 0). Because reaction was also found to type-specific monoclonal antibodies such as HC1, HC2, and HC3 (*1*), raised against gC of HHV-1, that virus was identified as the etiologic virus type. No cross-reactivity was observed with antisera against other species of herpesviruses, except a distinct reaction with a polyvalent anti-CeHV-1 antiserum due to the well-known cross-reactivity between HHV-1 and CeHV-1.

To discriminate between HHV-1 and HHV-2, type-specific PCR was performed according to the protocol of Piiparinen and Vaheri, using their published primers (*2*). Amplification products were detected in a 2% agarose gel stained with SYBR-Green. A 229-bp fragment was amplified, indicative of HHV-1 in the patient sample (lane S). Lanes 1 and 2 represent amplification products of HHV-1 strain Wal (229 bp) and HHV-2 strain D316 (241 bp), respectively (Figure).

Additionally, a multiplex PCR reaction detecting HHV1-6 (including HHV-3, also called Varicella-zoster virus 1; HHV-4, commonly known as Epstein-Barr virus; and HHV-5, human cytomegalovirus) was performed by using the primer setup described by Tenorio et al. (*3*). When these authors’ published set of primers was used, an HHV-1–specific fragment was also amplified (data not shown). Together with the reaction with different HHV-1–specific antibodies, including subtype-specific monoclonal antibodies, this was a clear indication of HHV-1 virus’s being the causative agent, excluding other possible primate herpesviruses.

## Discussion

The increasing number of pet monkeys kept in households in the United States has prompted concerns about the potential for transmission of the primate herpesvirus (formerly SHBV, now termed nonhuman primate virus or CeHV-1). Unlike the situation in the natural host, CeHV-1 can cause fatal encephalitis in humans. Persons working with certain macaque species may be at particular risk (*4*).

In this report, we describe the reverse problem—the growing evidence that human herpesviruses endanger other primates (*5*). Several nonhuman primate species have been reported to be susceptible to infection with human alphaherpesviruses. Experimental infections have been performed in owl monkeys, Cebus monkeys, Cotton-head Tamarins, and White-fronted Capuchins (*6*–*8*). Moreover, HHV-1 can naturally pass to primitive primates such as tree shrews (*9*).

In Old World primates, reports of human HHV-1 infections (*10*–*12*) indicate a virus-host relationship similar to that in humans, although sporadic fatal cases have been described, mainly in very young animals (*13*,*14*). In New World monkeys, however, HHV-1 seems to act more like a CeHV-1–type ”killer virus” (*5*,*15*). The case presented here is the third confirmed case of naturally transmitted HHV-1 infection in marmosets (*15*–*17*), and several other cases have been suspected to be of similar origin (*7*,*18*–*21*).

An outbreak of a fatal HHV-1 infection was observed recently in a group of common marmosets *(C. jacchus*) housed as a family group at the German Primate Center, Göttingen, Germany (K. Mätz-Rensing et al., unpub. data). All marmoset family members died within 3 days, indicating that HHV-1 has the capability to spread from monkey to monkey (K.D. Jentsch, pers. comm.).

We were not able to determine an HHV-1 history for the people in our case’s immediate surroundings, but a negative history would not be informative because asymptomatic shedding often contributes to HHV-1 transmission.

Because of the frequent but inapparent shedding of herpesviruses, direct contact of infected humans to animals should be limited. We suggest that keeping primates should be restricted to specialists, who are aware of the reciprocal hygienic risks. Use of gloves and eye protection, and even masks if aerosol transmission is suspected, is mandatory in standard biosafety precautions used in laboratory animal facilities. For more information, view the biosafety manual prepared by the Centers for Disease Control and Prevention and the National Institutes of Health at http://www.orcbs.msu.edu/biological/BMBL/BMBL-1.htm. Recommendations for precautions to be taken with any pet can be found at http://www.ahc.umn.edu/rar, http://www.cdc.gov/ncidod/op/pets.htm, and at http://www.cdc.gov/hiv/pubs/brochure/oi_pets.htm.

In contrast, increasing numbers of websites praise a vast variety of exotic pets and only rarely mention the potential health hazards (especially to children) from close contact or keeping pet monkeys in the household. Careful animal handling and certain hygienic restrictions should be strongly recommended for the sake of both owners and pet monkeys.
